# Oxidative stress’s impact on red blood cells: Unveiling implications for health and disease

**DOI:** 10.1097/MD.0000000000037360

**Published:** 2024-03-01

**Authors:** Emmanuel Ifeanyi Obeagu, Matthew Chibunna Igwe, Getrude Uzoma Obeagu

**Affiliations:** a Department of Medical Laboratory Science, Kampala International University, Ishaka, Uganda; b Department of Public Health, Kampala International University, Ishaka, Uganda; c School of Nursing Science, Kampala International University, Ishaka, Uganda.

**Keywords:** disease susceptibility, health implications, hematological disorders, public health interventions, red blood cells (RBCs), stress, stress-related RBC dysfunction

## Abstract

Oxidative stress, a condition characterized by an imbalance between reactive oxygen species (ROS) production and the body’s ability to detoxify them, has emerged as a pivotal factor in the pathophysiology of various diseases. Red blood cells (RBCs), essential components of the circulatory system, are particularly susceptible to oxidative damage due to their high oxygen-carrying capacity and the abundance of vulnerable biomolecules. This review comprehensively explores the intricate mechanisms underlying oxidative stress-induced damage to red blood cells and the subsequent implications for overall health and disease. We delve into the sources of ROS generation within RBCs, including metabolic processes and external factors, shedding light on the delicate redox balance that governs cellular homeostasis. The impact of oxidative stress on red blood cells extends beyond the confines of their primary physiological role, as these cells actively participate in immune responses, inflammation modulation, and nitric oxide metabolism. Consequently, understanding the implications of oxidative stress on RBCs provides valuable insights into the broader landscape of health and disease. In conclusion, this review underscores the critical role of oxidative stress in influencing red blood cell physiology and its far-reaching implications for human health. Elucidating the molecular intricacies of this relationship not only enhances our understanding of fundamental biological processes but also paves the way for the development of targeted therapeutic interventions to mitigate the adverse effects of oxidative stress on red blood cells and, by extension, on overall health.

## 1. Introduction

Stress, an inherent component of daily life, manifests as a physiological response to various environmental, psychological, or physiological challenges.^[1]^ Its pervasive influence extends beyond emotional states, impacting diverse facets of human health. Among the intricate physiological systems affected by stress, red blood cells (RBCs) stand as indispensable players in oxygen transport and tissue homeostasis.^[2]^ Emerging evidence suggests that stress, whether acute or chronic, intricately modulates the biology of these vital cellular entities, imposing consequential alterations that reverberate throughout the human body.^[3]^ The significance of red blood cells in maintaining oxygen equilibrium within the body cannot be overstated.^[4]^ These enigmatic cells, characterized by their biconcave discoid shape and hemoglobin-rich composition, ensure the delivery of oxygen to tissues and the removal of carbon dioxide, thereby supporting vital physiological functions. However, recent scientific endeavors have uncovered the susceptibility of RBCs to stress-induced changes, prompting a deeper exploration into the intricate relationship between stress and these essential blood components.^[5]^

The aim of this paper is to delineate the intricate interplay between stress and red blood cells. It sets the stage by elucidating the physiological underpinnings of stress as a fundamental adaptive response. Furthermore, it underscores the pivotal role of red blood cells in oxygen transport and outlines their vulnerability to stress-mediated modifications, encompassing alterations in morphology, function, and molecular composition. Moreover, the paper aims to highlight the burgeoning research that unravels the molecular mechanisms through which stress impinges upon red blood cells. These mechanisms encompass oxidative stress-induced damage, alterations in membrane dynamics, disruptions in hemoglobin structure and function, and perturbations in erythropoiesis, collectively shaping the intricate landscape of stress-mediated effects on RBC biology. This paper endeavors to comprehensively explore the far-reaching implications of stress on red blood cells, elucidating how these alterations may contribute to health and disease outcomes. By comprehensively understanding the intricate nexus between stress and RBCs, this review aims to underscore the significance of this relationship in clinical contexts and potentially unravel novel therapeutic avenues.

## 2. Stress

Stress refers to the body’s response to environmental, psychological, or physiological demands that challenge or exceed an individual’s adaptive capacities. It’s a natural physiological reaction aimed at coping with perceived threats or pressures, commonly known as stressors.^[6]^ When a person encounters a stressor, whether it’s a physical threat, an emotional challenge, or a significant life event, the body initiates a complex cascade of physiological responses. These responses involve the activation of the autonomic nervous system and the release of stress hormones such as cortisol and adrenaline, leading to a range of physical, emotional, and cognitive changes.^[7]^ Acute Stress occurs in response to immediate or short-term stressors. It’s the body’s rapid response to a perceived threat, often referred to as the “fight-or-flight” response. Acute stress is typically brief and can be beneficial in certain situations, helping individuals react quickly to challenges.^[8]^ Chronic stress arises from ongoing stressors or situations that persist over an extended period. It can result from various factors such as work-related pressures, financial difficulties, relationship issues, or chronic illness. Prolonged exposure to chronic stress can have detrimental effects on physical health, mental well-being, and overall quality of life.^[9]^ Chronic stress can contribute to various physical health problems, including cardiovascular issues, weakened immune function, digestive problems, headaches, muscle tension, and sleep disturbances.^[9]^

Stress can also impact mental health, leading to anxiety, depression, mood swings, irritability, difficulty concentrating, and reduced resilience in coping with challenges.^[10]^ Some individuals may adopt unhealthy coping mechanisms in response to stress, such as overeating or undereating, increased use of alcohol or drugs, social withdrawal, or changes in sleep patterns.^[11]^ While stress is a normal part of life, chronic or excessive stress can have detrimental effects on health and well-being. Managing stress effectively through healthy coping strategies like exercise, relaxation techniques, mindfulness practices, maintaining a balanced lifestyle, seeking social support, and seeking professional help when needed, is crucial for maintaining overall health and resilience in dealing with life’s challenges. Oxidative stress is a physiological condition characterized by an imbalance between the production of reactive oxygen species (ROS) and reactive nitrogen species (RNS) and the ability of the biological system to detoxify or repair the resulting damage. Reactive oxygen species and RNS are highly reactive molecules that contain oxygen or nitrogen and possess unpaired electrons, making them prone to interact with cellular components, such as lipids, proteins, and DNA. While these species play essential roles in various cellular processes, an excess of ROS and RNS can lead to detrimental effects, causing oxidative damage to biomolecules. Reactive oxygen species participate in cell signaling, immune responses, and defense mechanisms. However, excessive ROS production can lead to oxidative damage by oxidizing lipids (lipid peroxidation), proteins (protein carbonylation), and DNA (DNA strand breaks). Nitric oxide is involved in vasodilation, neurotransmission, and immune responses. However, overproduction of RNS, particularly peroxynitrite, can cause damage by nitrating proteins, altering their structure and function. Reactive oxygen species react with unsaturated fatty acids in cell membranes, leading to the formation of lipid peroxides. Reactive oxygen species and RNS modify amino acid residues in proteins, leading to protein oxidation. Reactive oxygen species and RNS induce DNA strand breaks, base modifications, and DNA-protein cross-links. Cumulative oxidative damage can activate signaling pathways leading to programmed cell death (apoptosis) or contribute to cellular senescence.^[10,11]^

## 3. Red blood cells

Red blood cells (RBCs), also known as erythrocytes, are specialized cells in the blood that play a vital role in oxygen transport throughout the body. They are produced in the bone marrow through a process called erythropoiesis and have a unique structure and function tailored for their essential task.^[12]^ RBCs are small, flexible, biconcave-shaped cells without a nucleus in humans (nucleated in some other species). This lack of a nucleus provides more space for the cell’s primary component, hemoglobin.^[13]^ The primary function of red blood cells is to transport oxygen from the lungs to various tissues and organs in the body and to carry carbon dioxide back to the lungs for elimination. This oxygen transport occurs via hemoglobin, a protein within RBCs that binds to oxygen in the lungs and releases it in tissues.^[14]^

Each RBC contains millions of hemoglobin molecules, which give blood its red color. Hemoglobin consists of 4 protein chains, each containing a heme group that can bind to oxygen. The iron within the heme group is essential for oxygen binding.^[15]^ Red blood cells have a remarkable capacity to carry oxygen due to the high concentration of hemoglobin. This enables efficient gas exchange in the lungs and tissues.^[13]^ The average lifespan of a red blood cell is about 120 days. After this period, aging or damaged RBCs are removed by the spleen and liver, and new RBCs are continuously produced in the bone marrow to maintain a stable number in circulation.^[11]^ Red blood cells also help maintain the body’s acid-base balance by transporting carbon dioxide, which can form bicarbonate ions, aiding in pH regulation.^[12]^ Disorders related to red blood cells include anemia (a condition characterized by a decrease in the number of RBCs or hemoglobin), polycythemia (an increase in the number of RBCs), and various inherited or acquired conditions affecting RBC structure or function.

Red blood cells (RBCs), also known as erythrocytes, are specialized cells that play a crucial role in oxygen transport throughout the body. In addition to hemoglobin, which is the primary oxygen-carrying molecule, RBCs possess various other components and participate in essential metabolic pathways to maintain their structure and function. Band 3 protein is a critical integral membrane protein that serves as an anion exchanger, facilitating the movement of chloride and bicarbonate ions across the RBC membrane. Glycophorins are glycoproteins present on the RBC membrane surface. The cytoskeleton of red blood cells mainly consists of spectrin, actin, and other associated proteins. Red blood cells rely predominantly on glycolysis for energy production since they lack a nucleus, mitochondria, and other organelles. Glucose is converted to pyruvate through a series of enzymatic reactions, generating ATP as the primary source of energy. ATP is essential for maintaining ion gradients, cell deformability, and other vital cellular functions. The Pentose Phosphate Pathway runs parallel to glycolysis and serves multiple functions, including the generation of NADPH and ribose-5-phosphate. Glucose-6-phosphate is diverted into the Pentose Phosphate Pathway, leading to the production of NADPH, which is crucial for protecting RBCs from oxidative stress. NADPH is involved in maintaining the cellular redox balance and supporting the activity of glutathione, an important antioxidant in RBCs. RBCs lack mitochondria, preventing oxidative phosphorylation and making glycolysis their primary source of ATP. Since RBCs operate under anaerobic conditions, glycolysis is the sole means of ATP production, and lactate is generated as the end product.^[11,12]^

## 4. Oxidative stress-induced changes in red blood cells

Stress can influence red blood cells (RBCs) in several ways, resulting in notable changes in their physiology, morphology, and function.^[16]^ Prolonged stress might lead to changes in RBC shape, affecting their flexibility and deformability. Stress-induced alterations can result in changes to the biconcave discoid shape, and leptocytes potentially impacting their ability to navigate through narrow capillaries and affecting oxygen delivery to tissues.^[17]^ Stress can trigger the production of reactive oxygen species (ROS) in the body, leading to oxidative stress. Increased oxidative stress can affect RBC membranes, causing lipid peroxidation and compromising membrane integrity.^[18]^ Chronic stress may impact hemoglobin’s structure and function within RBCs. Changes in hemoglobin conformation or oxygen-binding capacity can affect oxygen transport efficiency.^[19]^ Stress hormones, such as cortisol, can influence the bone marrow environment, potentially affecting erythropoiesis (the process of RBC production). Dysregulated erythropoiesis might lead to changes in RBC count or characteristics.^[20]^ Prolonged exposure to stress might shorten the lifespan of RBCs. Stress-induced changes could lead to premature aging or increased susceptibility to removal by the spleen, resulting in decreased RBC survival.^[21]^

Stress can trigger an inflammatory response in the body, influencing cytokine levels and immune function. Inflammatory mediators might impact RBCs directly or indirectly, affecting their function and survival.^[22]^ Stress-related alterations might impact RBC membrane dynamics, affecting its rigidity and permeability. Changes in membrane properties could impair the cell’s ability to maintain its structural integrity or perform its functions efficiently.^[23]^

The close relationship between red blood cells (RBCs) and the production of oxidant species stems from the inherent nature of RBCs as oxygen carriers and their continuous exposure to molecular oxygen during the respiratory process. This relationship is central to understanding the challenges and adaptations that red blood cells undergo to maintain their functionality. Despite the continuous exposure to molecular oxygen and the potential for oxidant species production, red blood cells have evolved intricate antioxidant defense mechanisms, including enzymes like superoxide dismutase and glutathione peroxidase, to counteract oxidative stress. However, under certain conditions, such as in hemolytic disorders or exposure to environmental toxins, the balance may be disrupted, leading to oxidative damage and potential implications for red blood cell function and overall health. Understanding this relationship is crucial for exploring the intricate interplay between oxygen transport and redox homeostasis in red blood cells.^[23]^

Stress-induced changes in red blood cells (RBCs) can lead to alterations in their structure and function, including the formation of methemoglobin. These changes are part of the body’s response to various stressors and may have implications for homeostasis. Stressors, such as exposure to environmental toxins, drugs, or pathological conditions, can lead to an increase in oxidative stress within the body. The stress response may trigger the production of reactive oxygen species (ROS) within RBCs, challenging their antioxidant defense mechanisms. Methemoglobin is a form of hemoglobin where the iron atom in the heme group is oxidized from the ferrous (Fe2+) to the ferric (Fe3+) state.^[22]^ Exposure to oxidative stress can lead to the oxidation of hemoglobin, resulting in the formation of methemoglobin. Certain drugs or chemicals, such as nitrites or aniline derivatives, can directly induce methemoglobin formation. Some individuals may have genetic mutations that predispose them to increased methemoglobin levels. Methemoglobin has a reduced ability to bind oxygen compared to normal hemoglobin. This can impair the oxygen-carrying capacity of RBCs. Increased levels of methemoglobin may lead to reduced oxygen delivery to tissues, potentially causing tissue hypoxia and compromising cellular function. The body has a built-in defense mechanism, known as the methemoglobin reductase system, which includes enzymes like NADH-dependent methemoglobin reductase. Methemoglobin reductase enzymes convert methemoglobin back to functional hemoglobin by reducing the iron from the ferric (Fe3+) to the ferrous (Fe2+) state. This enzymatic system helps maintain the balance between methemoglobin and functional hemoglobin, preventing excessive methemoglobin accumulation and preserving oxygen-carrying capacity. Excessive methemoglobin formation can lead to a condition known as methemoglobinemia, characterized by elevated levels of methemoglobin in the blood. Methemoglobinemia may present with symptoms such as cyanosis (bluish discoloration of the skin), shortness of breath, and fatigue. In severe cases, medical intervention may be required, including the administration of methylene blue, a compound that helps reduce methemoglobin to hemoglobin.^[23]^

## 5. Health implications of stress-induced RBC alterations

Stress-induced alterations in red blood cells (RBCs) can have significant implications for health, contributing to various physiological changes and potentially impacting overall well-being.^[24]^ Changes in RBC morphology, flexibility, or hemoglobin function due to stress-induced alterations can impair the efficient transport of oxygen to tissues. Reduced RBC deformability may hinder their ability to navigate through microcirculation, affecting oxygen delivery to organs and tissues. Chronic stress and its impact on RBC production or survival could contribute to the development or exacerbation of anemia. Stress-related alterations might lead to decreased RBC lifespan or dysregulated erythropoiesis, resulting in lower RBC counts or reduced hemoglobin levels.^[25]^ Impaired RBC function due to stress-induced changes might have implications for cardiovascular health. Reduced oxygen-carrying capacity or alterations in RBC dynamics could contribute to tissue hypoxia, potentially impacting heart function, circulation, and overall cardiovascular performance.^[26]^ Stress-related oxidative stress can affect RBC membranes and intracellular components. Increased production of reactive oxygen species (ROS) can lead to lipid peroxidation, compromising membrane integrity and potentially increasing RBC susceptibility to damage or premature destruction.^[2]^ Stress-induced changes in RBCs may influence immune responses and inflammatory processes. Altered RBC characteristics might affect interactions with immune cells or cytokine signaling, contributing to immune dysregulation or increased susceptibility to infections.^[2]^ Changes in RBC properties or altered hemostatic balance due to stress-related alterations could potentially contribute to an increased risk of thrombotic events. Abnormal RBC interactions or alterations in blood viscosity might influence clot formation and vascular function.^[27]^

## 6. Antioxidant capability of red blood cells

Red blood cells (RBCs) possess antioxidant capabilities that are essential for protecting themselves against oxidative stress and maintaining cellular integrity. While RBCs lack some of the typical cellular structures and organelles found in other cell types, they have evolved specific mechanisms to counteract the damaging effects of reactive oxygen species (ROS). RBCs contain SOD, an enzyme that catalyzes the dismutation of superoxide radicals (O2·−) into hydrogen peroxide and oxygen. RBCs also express catalase, an enzyme that converts hydrogen peroxide into water and oxygen.^[26]^ This helps prevent the accumulation of harmful levels of hydrogen peroxide. Glutathione is a crucial intracellular antioxidant present in RBCs. It acts as a reducing agent, directly neutralizing ROS and reactive nitrogen species (RNS). Glutathione also helps regenerate vitamin C, another important antioxidant. The methemoglobin reductase system, including enzymes like NADH-dependent methemoglobin reductase, plays a role in maintaining the redox state of hemoglobin. This system converts methemoglobin (Fe3+) back to functional hemoglobin (Fe2+), preventing the accumulation of nonfunctional methemoglobin. RBC membranes contain vitamin E, a lipid-soluble antioxidant that protects cell membranes from lipid peroxidation. Glutathione peroxidase, a selenium-containing enzyme, is present in RBCs and helps detoxify hydrogen peroxide and organic peroxides. The primary function of RBCs is to transport oxygen, and the binding of oxygen to hemoglobin helps prevent autooxidation of the iron in the heme group, reducing the potential for ROS generation. Since RBCs rely on anaerobic glycolysis for energy production and lack mitochondria, they produce fewer ROS compared to cells with oxidative metabolism. RBCs can scavenge nitric oxide, preventing its excessive consumption and contributing to the regulation of vascular tone.^[2]^

Antioxidant therapies play a crucial role in mitigating the progression of oxidative stress-related diseases, including those affecting red blood cells (RBCs). By neutralizing reactive oxygen species (ROS) and preventing oxidative damage, these therapies aim to restore redox balance and alleviate the impact of oxidative stress.^[27]^

## 7. Disease susceptibility and stress-related RBC dysfunction

Stress-related alterations in red blood cells (RBCs) may influence disease susceptibility and contribute to the pathophysiology of various health conditions.^[12]^ Chronic stress and its impact on RBC production, survival, or function could contribute to the development or exacerbation of anemia. Stress-related alterations might lead to decreased RBC counts, reduced hemoglobin levels, or impaired erythropoiesis, contributing to various types of anemia or hematological disorders.^[25]^ Stress-induced changes in RBC properties may affect oxygen transport efficiency, potentially contributing to tissue hypoxia. In cardiovascular diseases like ischemic heart disease or stroke, compromised RBC function due to stress-related alterations might exacerbate tissue damage or increase susceptibility to ischemic events.^[27]^

Stress can influence immune responses, and stress-related changes in RBCs might impact interactions with the immune system. Altered RBC characteristics might affect immune cell interactions, cytokine signaling, or inflammatory processes, potentially contributing to immune dysregulation or increased susceptibility to infections.^[28]^ While the direct relationship between stress-related RBC alterations and cancer/metabolic diseases is complex, chronic stress can impact physiological processes that influence disease progression. Stress-induced alterations in RBCs might contribute to an environment conducive to cancer growth or influence metabolic imbalances associated with conditions like diabetes or metabolic syndrome.^[25]^ Stress-related changes in RBC properties might affect blood viscosity, hemostatic balance, or RBC-endothelial interactions. These alterations could potentially contribute to an increased risk of thrombotic events or vascular disorders by influencing clot formation, endothelial dysfunction, or blood flow dynamics.^[13]^

## 8. Implications for clinical and health policy making

The implications of understanding stress-related alterations in red blood cells (RBCs) for clinical practice and health policy-making are multifaceted and hold significance in several domains:

## 9. Clinical implications

### 9.1. Improved patient management

Recognizing the impact of stress on RBC physiology can aid healthcare professionals in better managing conditions associated with RBC dysfunction. It allows for more tailored treatment approaches, including addressing anemia, hematological disorders, or cardiovascular complications with consideration for stress-related factors.

### 9.2. Enhanced diagnostic approaches

Understanding stress-induced changes in RBCs may lead to the identification of novel biomarkers or indicators of stress-related health conditions. These insights could contribute to more accurate diagnostic criteria or screening tools for various diseases associated with RBC alterations.

### 9.3. Personalized therapeutic strategies

Insight into stress-mediated RBC changes might lead to the development of targeted interventions aimed at mitigating or reversing stress-induced RBC dysfunction. Tailored therapeutic strategies, such as stress management techniques or medications targeting RBC function, could improve patient outcomes.

### 9.4. Health monitoring and risk assessment

Monitoring stress-induced alterations in RBC properties may serve as an adjunct in health monitoring and risk assessment. Integrating stress-related RBC parameters into health assessments could aid in identifying individuals at higher risk for certain health conditions.

## 10. Health policy implications

### 10.1. Guideline development

Incorporating stress-related RBC parameters into clinical guidelines could help standardize diagnostic criteria and treatment approaches for conditions influenced by RBC alterations. Health policies can advocate for updated guidelines that consider stress-related factors in disease management.

### 10.2. Public health interventions

Health policies focused on public health could emphasize stress management programs and interventions aimed at reducing stress-related health risks. These interventions might include education, stress reduction strategies, and promoting healthier lifestyles.

### 10.3. Research funding prioritization

Policymakers can allocate research funding toward further investigating the mechanisms underlying stress-induced RBC alterations. Investing in research could lead to the development of innovative therapies or interventions targeting stress-related RBC dysfunction.

### 10.4. Health education and awareness

Policies supporting health education initiatives can raise awareness among healthcare professionals and the general public regarding the impact of stress on RBCs and its potential implications for health. This increased awareness could lead to proactive health-seeking behaviors and early intervention.

## 11. Conclusion

The intricate interplay between stress and red blood cells (RBCs) unveils a compelling narrative of how physiological stressors intricately influence the function, structure, and lifespan of these vital blood components. Stress-induced alterations in RBCs encompass multifaceted changes, spanning morphology, function, and molecular dynamics, with far-reaching implications for human health and disease. The evolving understanding of stress-related modifications in RBCs highlights their significance as potential contributors to various health conditions, including anemia, cardiovascular diseases, immune dysregulation, and potential implications in cancer or thrombotic events. These alterations underscore the profound impact of stress on the body’s physiological balance and the intricate mechanisms underlying RBC physiology.

Moreover, the recognition of stress-mediated changes in RBCs offers a promising avenue for clinical advancements, personalized interventions, and health policy initiatives. This understanding provides healthcare professionals with insights for more tailored patient management strategies, refined diagnostic approaches, and the potential development of targeted therapies aimed at mitigating stress-induced RBC dysfunction. In essence, the convergence of stress physiology and red blood cell biology signifies a compelling frontier, offering insights that transcend conventional paradigms, with the potential to revolutionize clinical care, shape health policies, and pave the way for a holistic understanding of stress-related health implications at the cellular level.

## Author contributions

**Conceptualization:** Emmanuel Ifeanyi Obeagu.

**Methodology:** Emmanuel Ifeanyi Obeagu, Getrude Uzoma Obeagu.

**Supervision:** Emmanuel Ifeanyi Obeagu.

**Visualization:** Emmanuel Ifeanyi Obeagu.

**Writing – original draft:** Emmanuel Ifeanyi Obeagu, Matthew Chibunna Igwe, Getrude Uzoma Obeagu.

**Writing – review & editing:** Emmanuel Ifeanyi Obeagu, Matthew Chibunna Igwe, Getrude Uzoma Obeagu.
